# Left atrial to ventricular volume ratio and relation to fitness, cardiovascular risk factors, and diastolic function in healthy individuals: the HUNT Study

**DOI:** 10.1093/ehjimp/qyae028

**Published:** 2024-04-17

**Authors:** Sigbjorn Sabo, Havard Dalen, John Nyberg, Bjørnar Leangen Grenne, Even Olav Jakobsen, Bjarne Martens Nes, Ulrik Wisløff, Jon Magne Letnes

**Affiliations:** Department of Circulation and Medical Imaging, Norwegian University of Science and Technology, Prinsesse Kristinas gt. 3, PO Box 8905, 7491 Trondheim, Norway; Clinic of Cardiology, St. Olavs University Hospital, Prinsesse Kristinas gt. 3, PO Box 3250 Torgarden, 7006 Trondheim, Norway; Department of Circulation and Medical Imaging, Norwegian University of Science and Technology, Prinsesse Kristinas gt. 3, PO Box 8905, 7491 Trondheim, Norway; Clinic of Cardiology, St. Olavs University Hospital, Prinsesse Kristinas gt. 3, PO Box 3250 Torgarden, 7006 Trondheim, Norway; Department of Circulation and Medical Imaging, Norwegian University of Science and Technology, Prinsesse Kristinas gt. 3, PO Box 8905, 7491 Trondheim, Norway; Department of Circulation and Medical Imaging, Norwegian University of Science and Technology, Prinsesse Kristinas gt. 3, PO Box 8905, 7491 Trondheim, Norway; Clinic of Cardiology, St. Olavs University Hospital, Prinsesse Kristinas gt. 3, PO Box 3250 Torgarden, 7006 Trondheim, Norway; Department of Circulation and Medical Imaging, Norwegian University of Science and Technology, Prinsesse Kristinas gt. 3, PO Box 8905, 7491 Trondheim, Norway; Clinic of Cardiology, St. Olavs University Hospital, Prinsesse Kristinas gt. 3, PO Box 3250 Torgarden, 7006 Trondheim, Norway; Department of Circulation and Medical Imaging, Norwegian University of Science and Technology, Prinsesse Kristinas gt. 3, PO Box 8905, 7491 Trondheim, Norway; Clinic of Cardiology, St. Olavs University Hospital, Prinsesse Kristinas gt. 3, PO Box 3250 Torgarden, 7006 Trondheim, Norway; Department of Circulation and Medical Imaging, Norwegian University of Science and Technology, Prinsesse Kristinas gt. 3, PO Box 8905, 7491 Trondheim, Norway; Centre for Research on Exercise, Physical Activity and Health, School of Human Movement and Nutrition Sciences, University of Queensland, Brisbane, Queensland, Australia; Department of Circulation and Medical Imaging, Norwegian University of Science and Technology, Prinsesse Kristinas gt. 3, PO Box 8905, 7491 Trondheim, Norway; Clinic of Cardiology, St. Olavs University Hospital, Prinsesse Kristinas gt. 3, PO Box 3250 Torgarden, 7006 Trondheim, Norway

**Keywords:** cardiopulmonary exercise testing, diastolic dysfunction, left atrial volume

## Abstract

**Aims:**

Left atrial (LA) and ventricular (LV) remodelling is thought to be balanced in healthy individuals, and the LA end-systolic volume (LAV) to LV end-diastolic volume (LVEDV) ratio (LA:LV) could help discriminate between pathological and physiological LA enlargement. We aimed to assess LA:LV and its associations with age, sex, and cardiovascular risk factors HbA1C, body mass index (BMI), systolic blood pressure, and peak oxygen uptake (VO_2peak_). The association to measures of LV diastolic function and filling pressures were compared with LAV and LA reservoir strain.

**Methods and results:**

Cardiopulmonary exercise testing and measurement of risk factors 10 years apart and echocardiography at follow-up was performed in 1348 healthy adults [52% women, mean (SD) age 59 (12) years] prospectively included in a large population study. All risk factors were significantly associated with LA:LV in univariate analyses, while BMI and VO_2peak_ were significantly associated with LA:LV in adjusted models. A higher LA:LV was associated with increased odds ratio (OR) of diastolic dysfunction [OR (95% CI) 2.6 (2.1, 3.3)]. Measures of LV filling pressures were more closely associated with LA:LV than LAV and LA reservoir strain, but LA reservoir strain was more closely related to some diastolic function measures. In individuals with LAV > 34 mL/m^2^, the LA:LV explained 29% of variance in VO_2peak_ (*P* < 0.001).

**Conclusion:**

A higher LA:LV was associated with, and may improve, assessment of diastolic dysfunction and filling pressures. The LA:LV differentiates VO_2peak_ in individuals with enlarged LAV and may have a role in evaluating whether LA enlargement reflects pathology.

## Lay Summary

The size of the left forechamber, also known as the left atrium, is used as a criterion when looking for signs of heart failure. However, an enlarged left atrium is also commonly found in healthy individuals with hearts adapted to exercise training. In the healthy state, both the forechambers and the main chambers (ventricles) are thought to adapt together and enlarge in a balanced way. Therefore, we explored whether an enlarged ratio between the left atrium and the left ventricle was more tightly related to other signs of maladaptation than left atrial size alone. Our results show that the left atrial to ventricular ratio may have a role in distinguishing heart failure from healthy adaptation.

## Graphical Abstract

**Graphical abstract:**
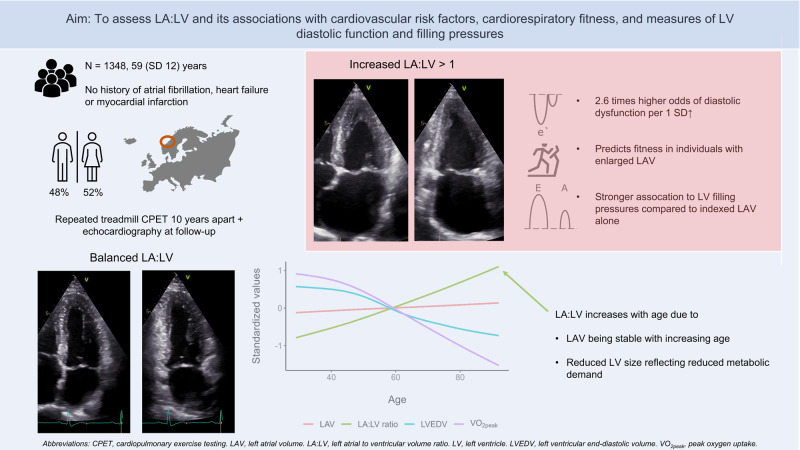

## Introduction

The left atrium (LA) plays a key role in maintaining normal cardiac function and supports left ventricular (LV) filling through its function as a reservoir, conduit, and pump.^1^ Therefore, LA and LV function are closely linked and adapt to meet specific metabolic demands.^2^ Cardiac chamber remodelling in response to pathology or altered physiologic stimuli often affects both chambers^3^ due to several pathophysiologic mechanisms.^4,5^ On the other side of the cardiovascular disease spectrum, chamber adaptations like LV hypertrophy and dilation may occur in response to exercise, representing a physiological pathway of remodelling.^6^

Left-sided chamber volumes are influenced by sex, age, body size, and other patient characteristics.^7,8^ Furthermore, both LV end-diastolic volume (LVEDV) and LA end-systolic volume (LAV) are positively associated with cardiorespiratory fitness.^9^ The LVEDV closely follows absolute peak oxygen uptake (VO_2peak_, L/min), and their ratio is maintained across fitness levels in healthy individuals,^9^ while LA size seems to be determined by a more complex interplay between cardiorespiratory fitness, LVEDV, and LV mass.^10^ Thus, LA enlargement is found both in patients with elevated filling pressures due to heart failure, including heart failure with preserved ejection fraction, and in athletes.^11,12^ However, the LV adapts differently in these two groups, with average to low LVEDV in heart failure with preserved ejection fraction opposed to a balanced increase in LVEDV relative to LAV in athletes. Thus, LA enlargement, defined in current guidelines as body surface area (BSA) indexed LAV >34 mL/m^2^,^13^ may not distinguish physiological adaptations from pathology. However, considering LA size in relation to LV size as the LAV to LVEDV ratio (LA:LV) could theoretically help to improve discrimination between pathologic and physiologic LA enlargement and support the often-challenging identification of diastolic dysfunction and elevated filling pressures.

In this study, we aimed to assess the interplay between remodelling of the LA and LV in a presumably healthy sample. Specifically, we aimed to evaluate the effect of age, sex, and cardiovascular risk factors on LA:LV. Secondly, we aimed to evaluate the relationship between LA:LV and conventional echocardiographic measures of diastolic function compared with LAV and LA reservoir strain.

## Methods

### Study sample

The third wave of the Trondelag Health study (HUNT3) was conducted between October 2006 and June 2008, inviting all citizens in the county of Nord-Trøndelag above 20 years, whereof 50 807 participants were included (54% of the eligible population). The HUNT3 Fitness Study, a sub-study of HUNT3, invited baseline participants in selected municipalities without cardiovascular, pulmonary, or malignant disease, sarcoidosis, or use of antihypertensive medication. A total of 4631 participants were included and completed cardiopulmonary exercise testing (CPET).^14^ Ten years later, HUNT4 was conducted with 56 042 participants in the baseline study. A total of 3264 baseline HUNT4 participants which also participated in the HUNT3 Fitness Study were invited to the HUNT4 Fitness and Echocardiography study, resulting in 1600 participants repeating CPET in HUNT4. Details regarding the HUNT Study cohorts have been published.^15^

**Figure 1:**
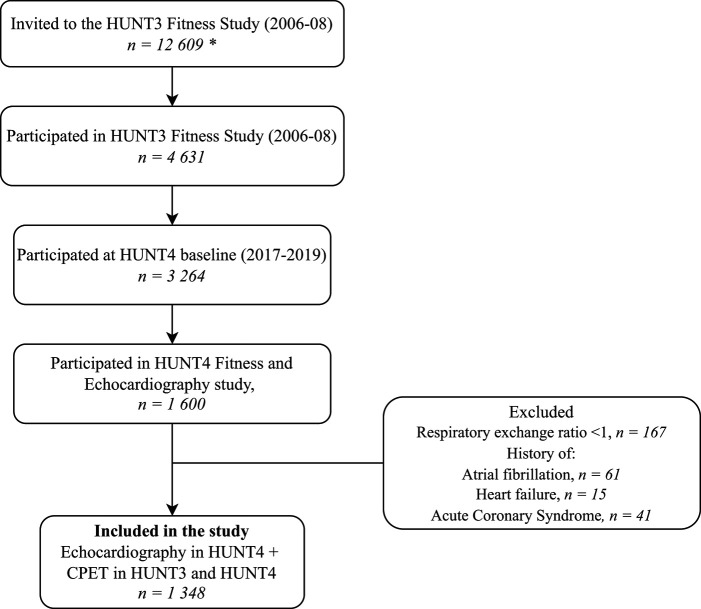
Flowchart of the study sample. *Fitness Study participants were recruited from the HUNT3 baseline study (2006–08) with 50 807 participants.

The data material of this study consist of participants from the HUNT3 Fitness Study and the combined Fitness and Echocardiography sub-study in HUNT4 (*Figure 1*), thus using both a cross-sectional and longitudinal prospective cohort design. Inclusion criteria were participation in the HUNT3 Fitness sub-study and the HUNT4 Fitness and Echocardiography sub-study. Exclusion criteria were (i) submaximal effort during CPET (defined as respiratory exchange ratio < 1.0), (ii) electrocardiogram-validated atrial fibrillation between HUNT3 and HUNT4 study inclusions, and (iii) presence of clinically validated heart failure or acute myocardial infarction between the HUNT3 and HUNT4 studies. The study was performed according to the Helsinki declaration and approved by the Regional Committee for Medical and Health Research Ethics of Mid-Norway (REC nr 13083).

### Cardiorespiratory fitness

VO_2peak_ was measured using the same treadmill CPET protocol in HUNT3 and HUNT4. Participants performed a stepwise protocol, increasing workload by higher treadmill inclination or speed every minute until voluntary exhaustion while wearing a tight face mask (Hans Rudolph, Germany) connected to a portable gas chamber analysis system (MetaMax II or MetaLyzer II; Cortex, Leipzig, Germany). VO_2peak_ was reported as absolute (L/min) or relative (mL/kg/min) values and was calculated as the average of the highest values from three 10 s measurements. Details regarding CPET in the HUNT Study have been published.^16^

### Echocardiography

All echocardiographic recordings were performed by one of two experienced sonographers and followed the study-specific protocol with guideline-directed specific chamber recordings aligned with the European Association of Cardiovascular Imaging recommendations.^17^ Recordings were performed using a Vivid E95 scanner (GE HealthCare, Horten, Norway) with a phased array transducer (M5S and 4V-D). Analyses were performed in EchoPAC (version 203–204; GE HealthCare). Echocardiographic recordings included at least three consecutive cardiac cycles.

LVEDV was measured by tracing the endocardial border in dedicated LV apical four-chamber and two-chamber recordings. The LV ejection fraction and biplane volumes were measured using Simpson’s method.^18^ LV mass was estimated using 2D linear measurements from the parasternal long-axis by the Cube formula,^18^ and LV concentric remodelling was defined as relative wall thickness > 0.42 and LV mass < 95 g/m^2^ for women and <115 g/m^2^ for men.^18^ LAV was quantified in LA focused apical two- and four-chamber views and calculated using the area-length and summation of disc methods. Indexed LAV was calculated using the formula for BSA of DuBois & DuBois, while LA:LV was calculated by dividing LAV by LVEDV.

LA strain was measured using the dedicated LA AFI package in LA focused apical two- and four-chamber views. The regions of interest were semi-automatically placed and manually adjusted to cover the LA wall. Tracking was visually evaluated, and the ROI width and position were adjusted, if necessary, in case of reduced tracking quality. Measurements were rejected in subjects where acceptable tracking was not achievable. All three LA strain parameters (reservoir, conduit, and contractile) were reported. According to the recommendation, the R-wave was used as reference, and thus, zero strain was set at the R-wave.^19^ Mitral inflow velocities (E- and A-wave), peak early mitral annular septal and lateral velocities (e′), peak tricuspid regurgitation (TR) velocity, and pulmonary vein systolic (S) and diastolic (D) velocities were measured in pulsed-wave and continuous Doppler recordings. Diastolic dysfunction was evaluated following recommendations from the American Society of Echocardiography and the European Association of Cardiovascular Imaging.^13^

### Statistical analyses

Descriptive data are presented as means and SDs or counts and proportions. Pearson’s correlations between LVEDV, LAV, and LV mass towards absolute VO_2peak_ (L/min) were investigated in 10-year age groups. Predictors of LA:LV were evaluated in univariable analyses using linear regression. Logarithmic transformation was applied for the LA:LV. Selected predictor variables were general participant characteristics [age, sex, systolic blood pressure, HbA1c, body mass index (BMI), relative VO_2peak_ (mL/kg/min), and diastolic function measures]. Multiple linear regression was used to evaluate the same predictor variables in age- and sex-adjusted models, as well as in a model adjusting for systolic blood pressure, HbA1c, and BMI. Predictor variables were from the HUNT4 study if not specified otherwise. Normality of residuals was evaluated using QQ- lots. The regression analyses for clinical variables and VO_2peak_ were also performed separately in participants with LAV > 34 mL/m^2^. Univariate linear regression analyses were performed to evaluate the association between LA:LV, LAV, and LA reservoir strain with measures of LV diastolic function and filling pressures, respectively. LAV, LVEDV, and LA:LV were standardized as deviance from the mean divided by the SD to show the relative relationship between volumes and ratio. For graphical illustration, a generalized additive model with integrated smoothness estimation was used to fit lines for the standardized values against age. A multiple logistic regression model was used to assess the relationship between diastolic dysfunction (yes/no) as the outcome with LA:LV as the predictor, and age, sex, systolic blood pressure, HbA1c, and BMI included as covariates. All statistical analyses were performed in R (R version 4.1.3,^20^ R Foundation for Statistical Computing, Vienna, Austria; R Studio^21^; packages ggplot^22^ and gtsummary^23^).

## Results

In total, 1348 participants with repeated CPET 10 years apart and echocardiography in HUNT4 were included in the study. General characteristics of the study sample are presented in *Table 1*. Mean age was 59 (±12, range 29–92) years, 52% were women, 39 (2.9%) were current smokers, and 159 (12%) were obese (BMI ≥ 30 kg/m^2^). Mean LVEDV was 111 (±31) mL and LV ejection fraction was 60 (±5) %. Mean LAV was 29.8 (±9.4) mL/m^2^ and LA:LV was 0.52 (±0.17). A total of 352 (26%) had LAV above 34 mL/m^2^, and 100 (7%) had echocardiographic evidence of diastolic dysfunction.

**Table 1:** General characteristics for the study sample at HUNT4

	Female, *N* = 697	Male, *N* = 651
Characteristic		
Age, years	58 (±12)	59 (±12)
Weight, kg	70 (±11)	85 (±11)
Height, cm	166 (±6)	179 (±6)
Body mass index, kg/m^2^	25.3 (±3.9)	26.4 (±3.1)
Body surface area, m^2^	1.77 (±0.14)	2.04 (±0.15)
Systolic blood pressure, mmHg	127 (±19)	132 (±16)
Diastolic blood pressure, mmHg	73 (±9)	78 (±10)
Peak oxygen uptake, mL/kg/min	34 (±8)	41 (±9)
Peak respiratory exchange ratio	1.11 (±0.05)	1.11 (±0.05)
Echocardiographic measures		
LV ejection fraction, %	60 (±4.8)	60 (±5.2)
LV indexed end-diastolic volume, mL/m^2^	54 (±11.7)	63 (±13.7)
LA indexed end-systolic volume, mL/m^2^	29 (±8.5)	31 (±10.0)
LA:LV volume ratio	0.55 (±0.2)	0.50 (±0.2)
LA reservoir strain	33.3 (±8.1)	32.4. (±8.2)
Diastolic dysfunction, *n* (%)	37 (±5.3%)	63 (±9.7%)
Mitral E-wave, cm/s	73 (±17)	67 (±16)
Mitral E/A	1.08 (±0.3)	1.16 (±0.3)
Lateral e′, cm/s	11 (±3.4)	11 (±3.5)
Septal e′, cm/s	8 (±2.5)	8 (±2.3)
Mitral deceleration time, ms	195 (±51)	214 (±64)
Pulmonary vein S, cm/s	63 (±13)	65 (±13)
Pulmonary vein S/D	1.35 (±0.4)	1.39 (±0.4)

Values are presented as mean (±SD) if not stated otherwise. bpm., beats per minute; HDL, high-density lipoprotein; LA, left atrial; LV, left ventricular; LA:LV, LA end-systolic volume to LV end-diastolic volume ratio.

**Figure 2:**
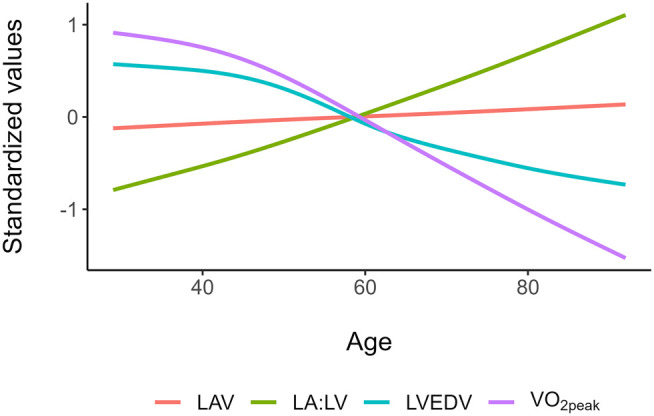
Standardized LAV, LVEDV, LA:LV, and VO_2peak_ vs. age. LAV, Left atrial end-systolic volume; LVEDV, Left ventricular end-diastolic volume; LA:LV ratio, LAV:LVEDV ratio; VO_2peak_, absolute peak oxygen uptake.

**Figure 3:**
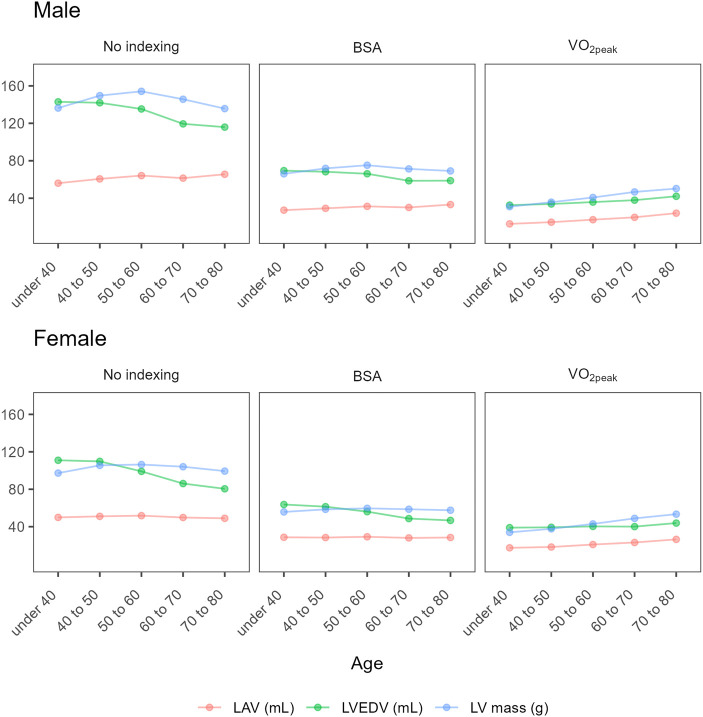
LAV, LVEDV, and LV mass without indexing and indexed against BSA and VO_2peak_. BSA, body surface area; LAV, left atrial volume; LV, left ventricle; LVEDV, left ventricular end-diastolic volume.

LVEDV decreased with higher age (*Figure 2*), and the correlation between LVEDV and absolute VO_2peak_ was strong in all 10-year age groups (see Supplementary data online, *Figure S1*). LVEDV followed absolute VO_2peak_ values in a balanced way with increasing age, keeping the LVEDV/VO_2peak_ ratio relatively unchanged (*Figure 3*). Age alone explained 11% of variance in LVEDV, but age was no longer significant after adjusting for absolute VO_2peak_. The LAV showed a weaker correlation with absolute VO_2peak_ than LVEDV across all age groups and was relatively unchanged with age, while the LAV/VO_2peak_ ratio and LA:LV were higher with higher age (*Figure 2*, *Table 2*). Age alone explained 12% of variance for LA:LV (*P* < 0.001), and there was no evidence of a non-linear effect of age. Women had significantly higher LA:LV than men (0.55 vs. 0.50, *P* < 0.001). Fifty-eight participants (4%) had concentric LV remodelling, and these had higher LA:LV values than those with normal geometry (0.61 vs. 0.52, *P* = 0.002).

**Table 2:** The mean LA:LV ratio with limits of normality two SDs below and above the mean by 10-year age groups and sex

Age	Female	Male
<40	0.46 (0.20, 0.71)	0.40 (0.21, 0.58)
40s	0.47 (0.22, 0.71)	0.43 (0.18, 0.69)
50s	0.53 (0.25, 0.81)	0.48 (0.19, 0.76)
60s	0.58 (0.24, 0.92)	0.52 (0.23, 0.81)
70s	0.62 (0.22, 1.00)	0.58 (0.22, 0.94)
80s	0.7 (0.15, 1.30)	0.65 (0.20, 1.10)

Values are mean (lower limit of normal, upper limit of normal). LA:LV, LA end-systolic volume to LV end-diastolic volume ratio.

Results from multiple regression analyses with LA:LV as the outcome variable are shown in *Table 3*. Age was the variable explaining the largest proportion of variance in LA:LV, although all variables including BMI, systolic blood pressure, HbA1c, relative VO_2peak,_ and measures of diastolic dysfunction showed significant associations in univariate regression analyses. Ten-year change in BMI was not significantly associated with LA:LV in the univariate model, but when adjusted for age and sex, it was positively associated with LA:LV. Ten-year change in systolic blood pressure showed a significant relationship in the univariate but non-significant relationship in the age- and sex-adjusted model (*Table 3*). Relative VO_2peak_ explained 9.5% of variance in LA:LV in the univariate model (*P* < 0.001) and was negatively associated with LA:LV. The explained variance was only modestly increased when VO_2peak_ was added to a model with age and sex (*R*^2^ without VO_2peak_ = 0.142; *R*^2^ with VO_2peak_ = 0.145, *P* = 0.033). When adjusted for age, sex, HbA1c, and systolic blood pressure as well, relative VO_2peak_ was still significantly associated with LA:LV, although now with a *higher* LA:LV [β (95% confidence interval (CI) 0.002 (0, 0.005) *R*^2^ = 0.187, *P* = 0.039]. In the subgroup of participants with LAV > 34 mL/m^2^, relative VO_2peak_ explained 29% of variance in LA:LV in univariate analysis, and vice versa, more than any of the other variables including age (see Supplementary data online, *Table S1*). In comparison, LA reservoir strain explained 16% of variance (*P* < 0.001). In the subgroup of participants with LAV > 34 mL/m^2^, LA:LV was significantly associated with relative VO_2peak_ after adjusting for age, sex, averaged e′, E/e′, elevated TR velocity, and LA reservoir strain (*P* < 0.001, model *R*^2^ = 0.58, *n* = 232). LA reservoir strain was not associated with VO_2peak_ in the same model (*P* = 0.6). Ten-year change in VO_2peak_ was not significantly associated with LA:LV in adjusted models for the total study sample (*Table 3*) nor for the subgroup with LAV > 34 mL/m^2^ (see Supplementary data online, *Table S1*).

**Table 3:** Regression analyses with log-transformed LA:LV as outcome variable

		Univariate			Age- and sex-adjusted			Adjusted		
Variable	*N*	β (95% CI)	*R* ^2^	*P*-value	β (95% CI)	*R* ^2^	*P*-value	β (95% CI)	*R* ^2^	*P*-value
Age	1159	0.009 (0.008, 0.011)	0.118	<0.001						
Sex (male)	1159	−0.09 (−0.126, −0.055)	0.020	<0.001						
HbA1c	1155	0.012 (0.007, 0.016)	0.023	<0.001	0.004 (0, 0.008)	0.144	0.07	0 (0.003, 0.003)	0.186	0.66^a^
Systolic BP	1158	0.003 (0.002, 0.004)	0.038	<0.001	0.002 (0.001, 0.003)	0.150	<0.001	0 (0, 0.002)	0.175	0.31^b^
Ten-year Δsystolic BP	1155	0.001 (0, 0.003)	0.004	0.017	0 (−0.001, 0.001)	0.139	0.68	0 (−0.002, 0.001)	0.170	0.59^b^
BMI	1157	0.017 (0.011, 0.022)	0.031	<0.001	0.019 (0.014, 0.024)	0.182	<0.001	0.018 (0.013, 0.023)	0.186	<0.001^a^
Ten-year ΔBMI	1155	0.001 (−0.009, 0.011)	−0.001	0.85	0.012 (0.003, 0.022)	0.146	0.011	0.010 (0, 0.020)	0.132	0.032^a^
VO_2peak_ (mL/kg/min)	1159	−0.011 (−0.013, −0.009)	0.095	<0.001	−0.003 (−0.006, 0)	0.145	0.033	0.003 (0, 0.006)	0.188	0.039^a^
Ten-year ΔVO_2peak_	1157	−0.26 (−0.396, −0.124)	0.011	<0.001	−0.04 (−0.172, 0.093)	0.141	0.56	0.02 (−0.11, 0.15)	0.185	0.76^a^

BP, blood pressure; BMI, body mass index; VO_2peak_, peak oxygen uptake.

^a^Adjusted for age, sex, systolic blood pressure, HbA1c, and BMI.

^b^Adjusted for current use of antihypertensive drugs, sex, HbA1c, and BMI.

### Associations of LA:LV and LAV with diastolic function measures

LA:LV was significantly associated with increased risk of diastolic dysfunction in multiple logistic regression analyses with an odds ratio (OR) (95% CI) of 2.6 (2.1, 3.3) per SD increase, adjusted for age, sex, systolic blood pressure, HbA1c, and BMI (*P* < 0.001). Furthermore, measurements of diastolic function including LA strain indices were significantly associated with LA:LV in univariate analyses (*Table 4*). Higher E, e′, and LA reservoir strain were all associated with lower LA:LV, while other parameters (E/e′, TR velocity, PV S/D ratio, LA conduit, and contractile strain) were associated with higher LA:LV. The *R*^2^ was highest for e′ and the E/e′ ratio (both *R*^2^ = 0.089 *P* < 0.001). Analyses with LAV as the outcome variable showed significant positive associations for E/e′, TR velocity, and LA reservoir and contractile strain (*Table 4*), but *R*^2^ were lower (highest *R*^2^: 0.027, LA contractile strain) compared with the LA:LV models. LA reservoir strain showed a higher *R*^2^ for e′ and E velocity, but not for E/e′ and TR velocity (*Table 4*). In a model with E/e′ as the outcome including LA:LV and LA reservoir strain as predictors, adjusting for age and sex, only LA:LV was significantly associated with E/e′ (*P* < 0.001 for LA:LV, *P* = 0.23 for LA reservoir strain, model *R*^2 =^ 0.19). Similarly, LA:LV significantly (*P* < 0.036) impacted TR velocity adjusted for age, sex, and LA reservoir strain (*P* = 0.86), but explained variance was still low (*R*^2^ = 0.025).

**Table 4:** Univariate regression models with diastolic function indices as outcome variables and LA:LV or indexed LAV as predictors

	LA:LV				LAV				LA reservoir strain			
Outcome	*N*	β (95% CI)	*R* ^2^	*P*-value	*N*	β (95% CI)	*R* ^2^	*P*-value	*N*	β (95% CI)	*R* ^2^	*P*-value
e′ averaged	1125	−2.64 (−3.14, −2.15)	0.088	<0.001	1192	−0.3 (−0.76, 0.15)	0.001	0.20	963	0.17 (0.15, 0.19)	0.256	<0.001
E velocity	1109	−3.85 (−7.01, −0.69)	0.004	0.017	1174	2.46 (−0.36, 5.28)	0.002	0.088	951	0.8 (0.68, 0.92)	0.150	<0.001
E/e′	1091	2.03 (1.64, 2.42)	0.085	<0.001	1156	0.62 (0.26, 0.99)	0.009	<0.001	934	−0.05 (−0.07, −0.04)	0.051	<0.001
TR velocity	493	0.18 (0.09, 0.27)	0.027	<0.001	511	0.16 (0.08, 0.24)	0.027	<0.001	421	0 (−0.01, 0)	0.005	0.072
PV S/D ratio	1016	0.27 (0.2, 0.34)	0.051	<0.001	1071	−0.03 (−0.09, 0.04)	0.000	0.46	871	−0.01 (−0.01, −0.01)	0.035	<0.001
LA reservoir strain	899	−6.25 (−7.95, −4.55)	0.054	<0.001	958	−1.05 (−2.61, 0.5)	0.001	0.18				
LA conduit strain	899	4.46 (3.02, 5.9)	0.039	<0.001	958	−0.79 (−2.09, 0.51)	0.000	0.23				
LA contractile strain	899	1.79 (0.77, 2.81)	0.012	<0.001	958	1.84 (0.93, 2.75)	0.015	<0.001				

LA, left atrium; LA:LV, left atrial to ventricular volume ratio; LAV, left atrial volume; PV, pulmonary vein; *R*^2^, coefficient of determination; S/D, systolic/diastolic; TR, tricuspid regurgitant velocity.

Regression models with LA:LV as the outcome variable and verified diastolic dysfunction as the explanatory variable showed statistically significant positive associations [univariate β (95% CI) 0.33 (0.27, 0.39), *R*^2^ = 0.08; adjusted model β (95% CI 0.29 (0.21, 0.33), both *P* < 0.001]. Explained variance in the univariate analysis was 8% (*R*^2 =^ 0.08) and contributed beyond the other included measures in the adjusted model (adjusted model with diastolic dysfunction *R*^2^ = 0.24; without diastolic dysfunction *R*^2^ = 0.19).

## Discussion

In this presumably healthy sample, the LA:LV volume ratio increased with higher age mainly attributed to lower LVEDV with higher age. This finding was closely associated with a decline in absolute VO_2peak_, while the LAV remained relatively unchanged with higher age. The LA:LV was associated with unfavourable levels of cardiovascular risk factors, and measures reflecting higher LV filling pressure (E/e′) and systolic pulmonary artery pressure (TR velocity) were positively associated with LA:LV. The association for LA:LV was stronger than for LAV and LA reservoir strain. Furthermore, LA:LV differentiated cardiorespiratory fitness levels in individuals with enlarged LAV, and a higher LA:LV was associated with an increased OR for the presence of diastolic dysfunction.

### Differences in LA and LV remodelling

LA:LV showed a modest association with relative VO_2peak_. LVEDV is closely linked to VO_2peak_, with a robust association across the age span.^24,25^ With higher age, lean body mass is reduced which is associated with reduction in energy expenditure (at rest and during exercise), and levels of exercise declines. Consequently, the metabolic demand decreases, as reflected by a decrease in absolute VO_2peak_. This is supported by descriptive data from Molmen *et al.*^26^ showing a preserved LVEDV/VO_2peak_ ratio despite changes in absolute VO_2peak_ in older adults. Conversely, our results suggest that LA size behaves more static across adulthood. One possible explanation for this phenomenon may be that the LAV adapts to different stimuli such as exercise or increased filling pressures but has less ability to reversely remodel with, e.g. reductions in exercise volumes. The LA has been shown to adapt to exercise volumes over shorter time spans,^27^ but this may not be true after long-term exercise stimuli. Possible explanations may be the anatomical constraints from pulmonary vein insertions with less adaptive atrial myocardium and a thinner wall with less myocardium than the LV. This leads to sustained LAV despite decreased LVEDV, causing increased LA:LV with higher age, as shown in the presented results.

### Association with diastolic function

TR velocity and E/e′ were significantly associated with increased LA:LV, likely explained by increased LV filling pressures with upstream increased LA pressures and following LA enlargement.^28,29^ The LA:LV explained more of the variance in measures of diastolic function than LAV, suggesting that the LA:LV may be a better measure of LV diastolic function and increased LV filling pressure than LAV alone and LA reservoir strain. This relationship for LA:LV was still significant after adjustment for both LA reservoir strain and LAV. However, LA reservoir strain correlated considerably better with both e′ and mitral E velocity than LA:LV. This might be due to that mitral E velocities are lowest with impaired relaxation of the LV and higher both with better and worse LV diastolic function. Furthermore, the study sample was predominantly healthy participants. Higher LA:LV was significantly associated with diastolic dysfunction in multiple logistic regression analysis. Thus, the LA:LV could hold merit in assessment of LV diastolic dysfunction and filling pressures to reduce ambiguity from enlarged LAVs. This is of importance, as the current algorithm for assessment of LV diastolic dysfunction leaves a considerable proportion of examinations as ‘indeterminate’.^30^ This way of assessing LA size also circumvents some of the challenges related to indexing of the LA,^31^ benefiting from the fact that the LV is tightly associated with the absolute VO_2peak_ in the healthy state.^9^ LA:LV explained 29% of variance in relative VO_2peak_ in those with an LAV > 34 mL/m^2^ and was also significantly associated with relative VO_2peak_ after adjusting for other diastolic function measures including LA reservoir strain. This supports the link to diastolic dysfunction and that the LA:LV could assist in interpreting whether an enlarged LAV is caused by physiological or pathological mechanisms.

### Impact of cardiovascular risk factors on LA:LV

In univariate analyses, systolic blood pressure, HbA1c, and BMI were all significantly and positively associated with LA:LV. These associations align with previous studies, as hypertension is a common cause of diastolic dysfunction,^32^ and LA enlargement is a frequent finding in obese as well as diabetic patients.^33^ Spevack *et al.*^34^ found that an increased LA to LV diameter ratio was associated with reduced exercise capacity, also when adjusted for age. They also reported an increase in this ratio with higher age. Their study sample included patients with a clinical indication for stress echocardiography, signifying a different population than the present study. McNamara *et al.* randomly allocated healthy, middle-aged adults to high-intensity exercise or stretching/balance control. They reported balanced LA and LV remodelling after 10 months of exercise intervention.^35^ However, after 24 months of exercise intervention, they observed a 32% increase in LA:LV from baseline, indicating greater LA than LV remodelling. Our results show that relative VO_2peak_ was significantly positively associated with LA:LV in adjusted analyses, similar to the finding by McNamara *et al.*^35^ of long-term exercise increasing the LA:LV. This finding somewhat contradicts our hypothesis, expecting a lower LA:LV with higher VO_2peak_. However, the effect size was small, and adjusting for possible mediators in the causal relationship between VO_2peak_ and LA:LV such as HbA1c, systolic blood pressure, and BMI could be the explanation behind the reversed association.

### Strengths and limitations

The main strengths of this study are (i) the large cohort consisting of healthy participants, (ii) clinical measures and gold standard measurements of cardiorespiratory fitness 10 years apart, and (iii) guideline-directed chamber-specific echocardiographic recordings. However, the study also has some limitations. As individuals with cardiovascular disease were excluded from these analyses, the association of VO_2peak_ with LA:LV may be underestimated. On the other hand, we found that in individuals with LAV > 34 mL/m^2^, LA:LV was strongly associated with relative VO_2peak_ and explained 29% of the variance. Voluntary participation in exercise testing and echocardiography implies a risk of selection bias. The primarily Caucasian study population may challenge the generalizability of the findings. Furthermore, the effect of long-term fitness on cardiac remodelling cannot be thoroughly evaluated as echocardiography was available only at follow-up. Finally, the relationship to LV filling pressures should ideally be proved using invasive haemodynamic assessments.

## Conclusion

A relatively unchanged LAV throughout adulthood accompanied by a decreasing LVEDV leads to a higher LA:LV volume ratio with age. A higher LA:LV is associated with unfavourable levels of cardiovascular risk factors. Measures indicating higher LV filling pressure were more closely associated with LA:LV than with LAV and LA reservoir strain, indicating that LA:LV has the potential to improve the assessment of LV diastolic dysfunction and filling pressures. The ability of the LA:LV to differentiate cardiorespiratory fitness levels in individuals with enlarged LAV implies that this ratio may have a role in evaluating whether LA enlargement reflects physiology or pathology. Future studies should compare LA:LV, LAV, and LA strain with respect to prediction of clinical outcomes.

## Supplementary Material

qyae028_Supplementary_Data

## Data Availability

The data from HUNT (Nord-Trøndelag Health Study) used in this study are available on application to the HUNT Data Access Committee in accordance with the policy on data availability (further information and contact information: https://www.ntnu.edu/hunt/data).

## Lead author biography

Sigbjørn Sæbø is a final year medical student. During his medical degree, he has completed the medical student research programme, performing studies evaluating artificial intelligence in echocardiography. After graduating, he will finalize his PhD before starting clinical training. For his medical thesis, he analysed data from the HUNT Study (a prospective longitudinal epidemiological study) and studied the relationship between cardiorespiratory fitness and measures of diastolic function.
