# Supplementary material for: Left atrial to ventricular volume ratio and relation to fitness, cardiovascular risk factors, and diastolic function in healthy individuals: the HUNT Study
Source: Eur Heart J Imaging Methods Pract. 2024 Apr 17;2(1):qyae028. doi: 10.1093/ehjimp/qyae028 (PMC11472746; doi:10.1093/ehjimp/qyae028)
Supplement: qyae028_Supplementary_Data [file qyae028_Supplementary_Data.docx]

**Supplemental Table 1.** Regression model analyses with log transformed LA:LV as outcome variable in participants with indexed left atrial end-systolic volume >34 mL/m^2^.

|  | | Univariate | | | Age- and sex-adjusted | | | Adjusted | | |
| --- | --- | --- | --- | --- | --- | --- | --- | --- | --- | --- |
| Variable | N | β (95% CI) | *R*^2^ | p-value | β (95% CI) | *R*^2^ | p-value | β (95% CI) | *R*^2^ | p-value |
| Age | 337 | 0.009 (0.007, 0.011) | 0.204 | <0.001 |  |  |  |  |  |  |
| Sex (male) | 337 | -0.094 (-0.142, -0.046) | 0.040 | <0.001 |  |  |  |  |  |  |
| HbA1c | 335 | 0.016 (0.01, 0.023) | 0.063 | <0.001 | 0.007 (0.001, 0.013) | 0.289 | 0.029 | 0.006 (0, 0.012 | 0.304 | 0.052* |
| Systolic BP | 337 | 0.001 (0, 0.003) | 0.011 | 0.029 | -0.001 (-0.002, 0) | 0.281 | 0.163 | 0 (-0.002, 0.001) | 0.315 | 0.371# |
| Ten-year ∆Systolic BP | 336 | 0.001 (-0.001, 0.002) | -0.001 | 0.456 | -0.001 (-0.002, 0) | 0.283 | 0.072 | 0 (-0.002, 0.001) | 0.313 | 0.529# |
| BMI | 337 | 0.008 (0, 0.015) | 0.009 | 0.047 | 0.009 (0.003, 0.016) | 0.293 | 0.006 | 0.009 (0.002, 0.015) | 0.304 | 0.009* |
| Ten-year ∆BMI | 336 | -0.003 (-0.017, 0.011) | -0.003 | 0.711 | 0.009 (-0.003, 0.021) | 0.281 | 0.132 | 0.010 (0.001, 0.013) | 0.294 | 0.104* |
| VO_2peak_ (mL/kg/min) HUNT4 | 337 | -0.013 (-0.015, -0.011) | 0.289 | <0.001 | -0.008 (-0.011, -0.005) | 0.322 | <0.001 | -0.007 (-0.011, -0.003) | 0.326 | <0.001* |
| Ten-year ∆VO_2peak_ | 336 | -0.334 (-0.499, -0.17) | 0.043 | <0.001 | -0.121 (-0.273, 0.031) | 0.281 | 0.118 | -0.092 (-0.244, 0.06) | 0.303 | 0.236* |

#Adjusted for current use of antihypertensive drugs, sex, HbA1c and BMI. *Adjusted for age, sex, systolic blood pressure, HbA1c and BMI. Abbreviations: BP, Blood pressure; BMI, Body mass index; VO_2peak_, Peak oxygen uptake.


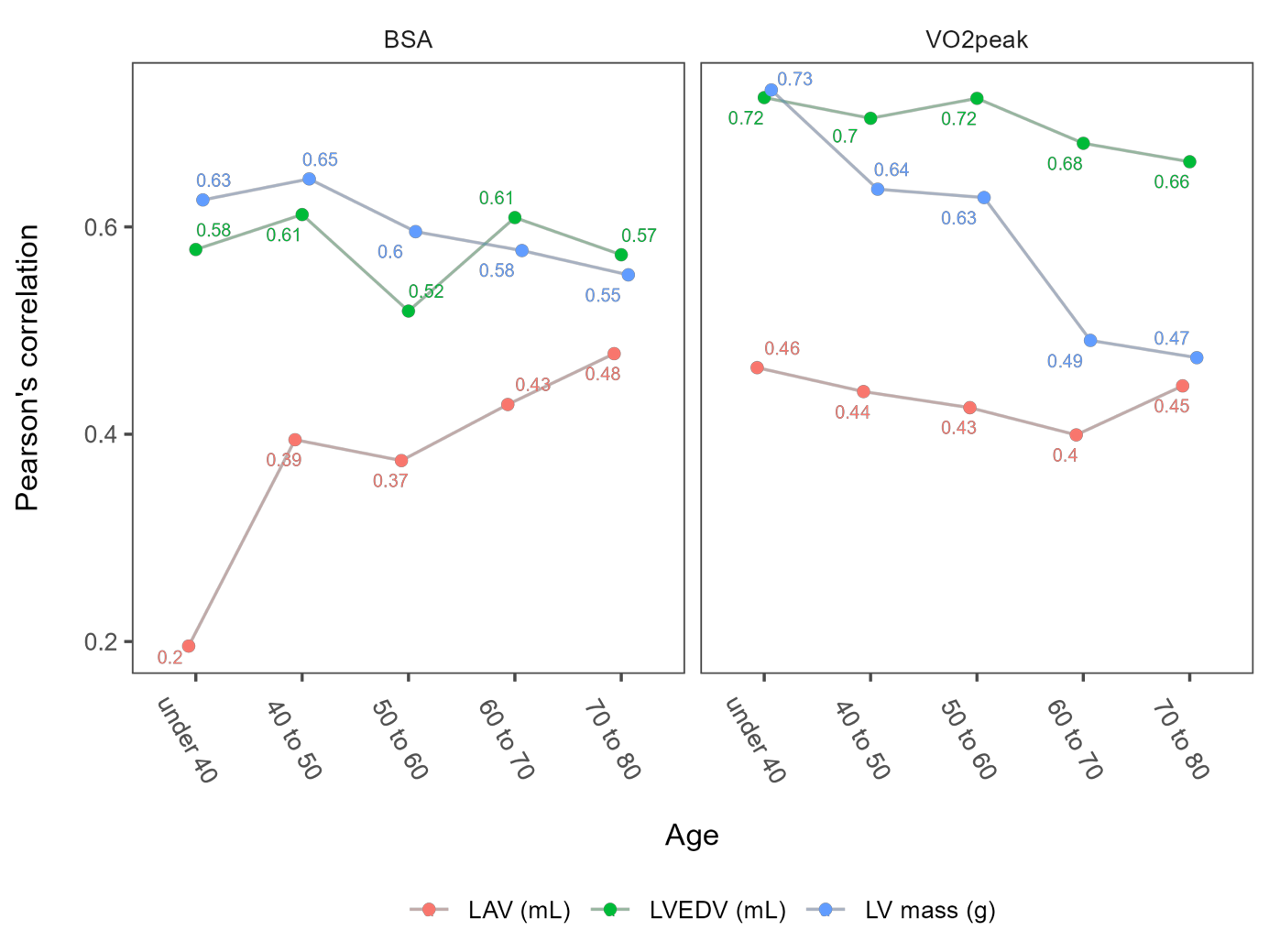


**Supplemental Figure 1.** Pearson’s correlation between indexation variables (BSA and absolute VO_2peak_) and LAV, LVEDV and LV mass. All correlations were significant with p <0.001, except for LAV vs. BSA in age group under 40 (p=0.105). Abbreviations: BSA, body surface area; LAV, left atrial volume; LV, left ventricle; LVEDV, left ventricular end-diastolic volume.
